# Supplementary material for: The normal human lymph node cell classification and landscape defined by high-dimensional spatial proteomics
Source: PLoS One. 2026 Apr 21;21(4):e0346693. doi: 10.1371/journal.pone.0346693 (PMC13099103; doi:10.1371/journal.pone.0346693)
Supplement: S1 File — (PDF) [file pone.0346693.s001.pdf]

SUPPLEMENTARY FIGURES

Title:
The normal human lymph node cell classification and landscape defined by high-dimensional spatial proteomics.

Short title: Lymph node landscape and cell classification

Authors:
Maddalena M Bolognesi[1,2 ¶], Lorenzo Dall'Olio[3 ¶], Giulio Eugenio Mandelli[4,5], Luisa Lorenzi[4,5], Francesca M Bosisio[6,7,8], Ann M Haberman[9,10], Govind Bhagat[11#], Simone Borghesi[12], Mario Faretta[13], Gastone Castellani[14 ¶] and Giorgio Cattoretti[15 ¶*]

Affiliations:
1 Istituto di Bioimmagini e Sistemi Biologici Complessi (IBSBC) – CNR Via F.lli Cervi, 93 20090 Segrate (MI).
2 NBFC, National Biodiversity Future Center, Palermo 90133, Italy.
3 Laboratorio di Data Science and Bioinformatics, IRCCS Istituto delle Scienze Neurologiche di Bologna – AUSL BO Ospedale Bellaria, Via Altura 3 - 40139 Bologna - Italy.
4 Pathology Unit, Department of Molecular and Translational Medicine-DMMT, University of Brescia, via Branze, 43, 25123 Brescia, Italy.
5 Pathology Unit, ASST Spedali Civili Di Brescia, Brescia,Italy.
6 The Leuven Institute for Single-cell Omics (LISCO), KULeuven, Leuven, Belgium.
7 Translational Cell and Tissue Research Unit, Department of imaging and pathology, KULeuven, Leuven, Belgium.
8 Pathology Department, University Hospital of Leuven, Leuven, Belgium.
9 Department of Immunobiology, Yale University, New Haven, Connecticut, USA.
10Department of Laboratory Medicine, Yale University, New Haven, Connecticut, USA.
11 Pathology, Columbia University Irving Medical Center and New York Presbyterian Hospital, NewYork, NY, USA.
12 Department of Mathematics and Applications, University of Milano Bicocca, 20126 Milan, Italy.
13 Department of Experimental Oncology, European Institute of Oncology IRCCS, 20139 Milan, Italy
14 Department of Experimental, Diagnostic and Specialty Medicine, University of Bologna, 40127 Bologna, Italy.
15 Pathology, Department of Medicine and Surgery, Universitá di Milano-Bicocca, 20900 Monza (MI), Italy.

¶ These authors contributed equally to this work
# present address: Pathology, Saint Louis University School of Medicine, Saint Louis, MO, USA

*Correspondence: giorgio.cattoretti@unimib.it

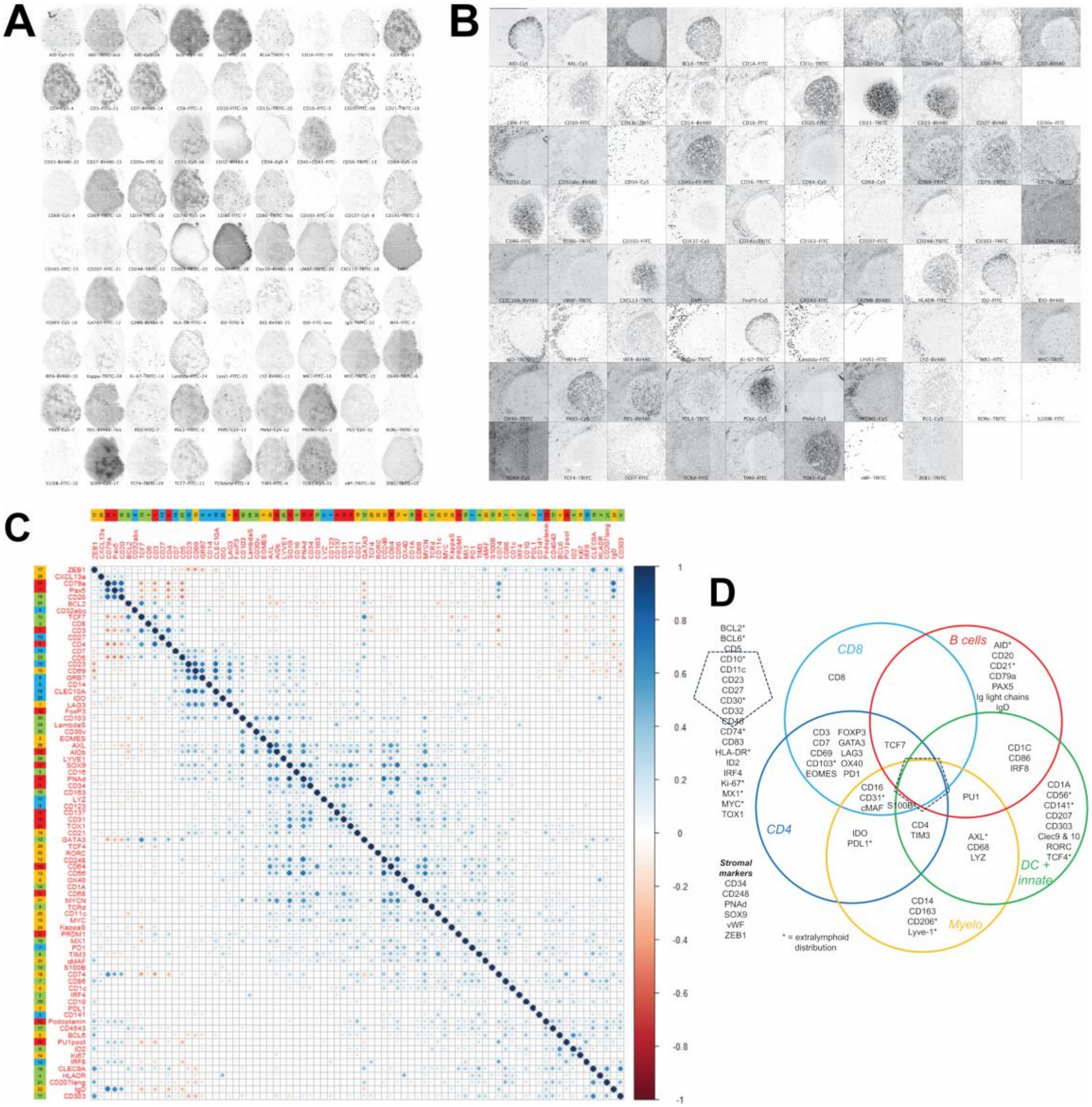

S1 Fig. (related to Fig 2). Representative IF images, antibody correlation and cell type distribution.

A: Low-power images of a whole LN (UPN106) stained for 81 markers. Each 8-bit image was enhanced via the Enhance-Contrast command in Fiji (0.35% saturated pixels). In each image, the biomarker, the fluorochrome and the round number is shown at the bottom. Note duplicate fluorochrome images for some markers. Scale bar 5 mm.

B: high-power magnification of 78 images from A, used for the analysis. In each image, the biomarker and the fluorochrome is shown at the bottom. Scale bar 500 µm.

C: Bivariate correlation matrix of all markers. Each marker is correlated against all others and the result plotted according to the scale on the right. On top and on the left the fluorochrome for imaging (blue: BV480; green: Alexa 488; orange: Rhodamine RedX; red: Alexa 647) and the staining round number are listed for each marker. Data is from UPN107. Image plot representing the correlation matrix of all markers (Antibodies), one towards all, obtained with the "corplot" function in R (reordered according to AOE, the angular order of the eigenvectors). Divergent color palette shows in blue positive correlation, in red negative correlation. Dot size

corresponds to the value of correlation. Notably, T-cell and B-cell markers clusterize separately in the table (top left corner).

D: Graphic representation of labeling across cell types for the panel of biomarkers used in this work. The colored circles represent CD4, CD8, B-cells (including plasma cells), innate immune cells (cDC, pDC, ILC3) and myelomonocytic cells. At the center, an inverted pentagram represents multiple lineage co-expression and is reported for clarity at the left of the Voronoi graph. Markers names are allocated according to the cell type distribution. The assignment is approximative. For additional information see Supplementary Table 2.

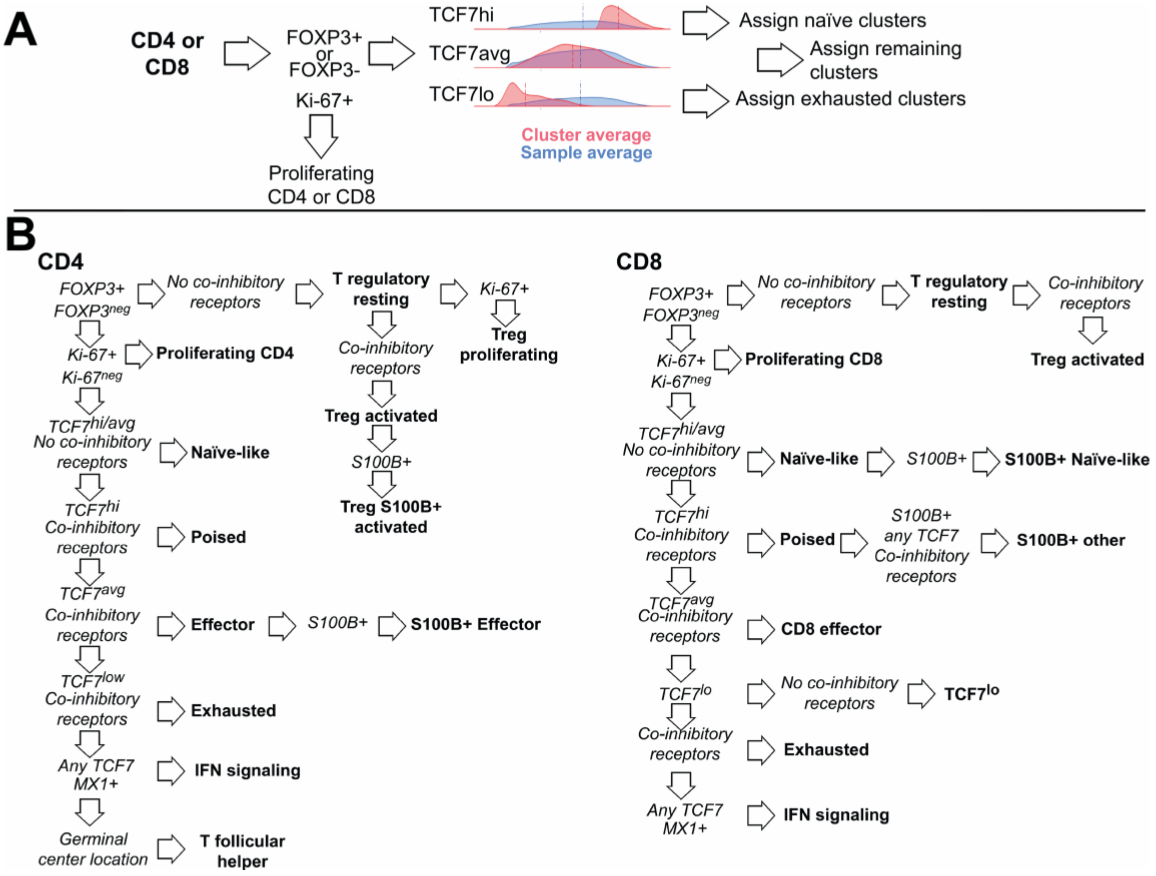

S2 Fig. (related to Fig 3). Flowcharts for the definition of T-cell subsets.

A: Flowchart for the assignment of the TCF7 level category. Non-proliferating CD4 or CD8 T-cells are classified based on high, low and intermediate levels of TCF7, as depicted by the cluster signal intensity profile (red), compared to the sample population (blue).

B: Flowchart for the assignment of the subset type for CD4 and CD8 T-cell clusters upon subclustering. The presence/absence of subclassifying markers is drawn from the BRAQUE ranked markers output (see[1, 2])

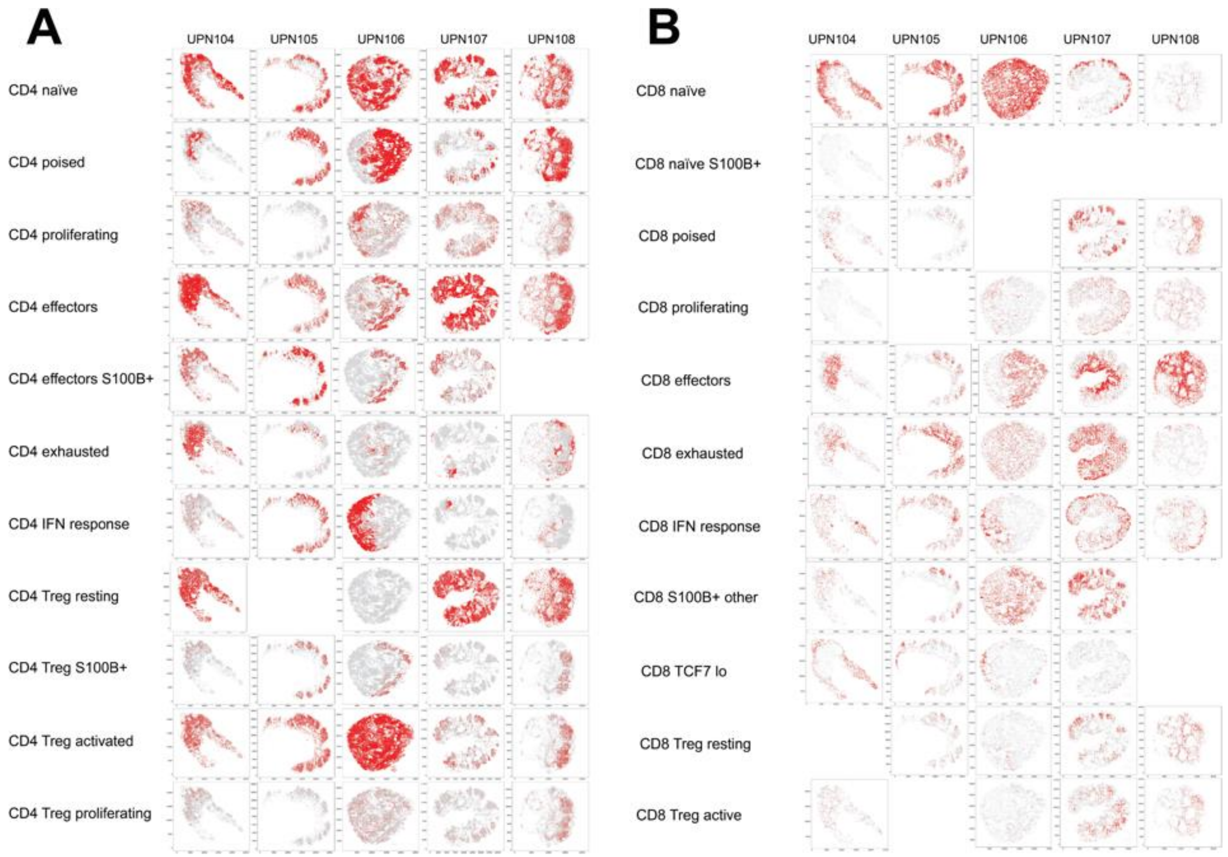

S3 Fig. (related to Fig 5). Spatial distribution of CD4 and CD8 T-cells.

A: Spatial distribution of CD4 T-cell subsets on five whole LN. Each population, the sum of the individual clusters, is shown in red over a gray shadow representing all CD4 T-cells. Each dot is a magnified cell.

B: Spatial distribution of CD8 T-cell subsets on five whole LN. Each population, the sum of the individual clusters, is shown in red over a gray shadow representing all CD8 T-cells. Each dot is a magnified cell.

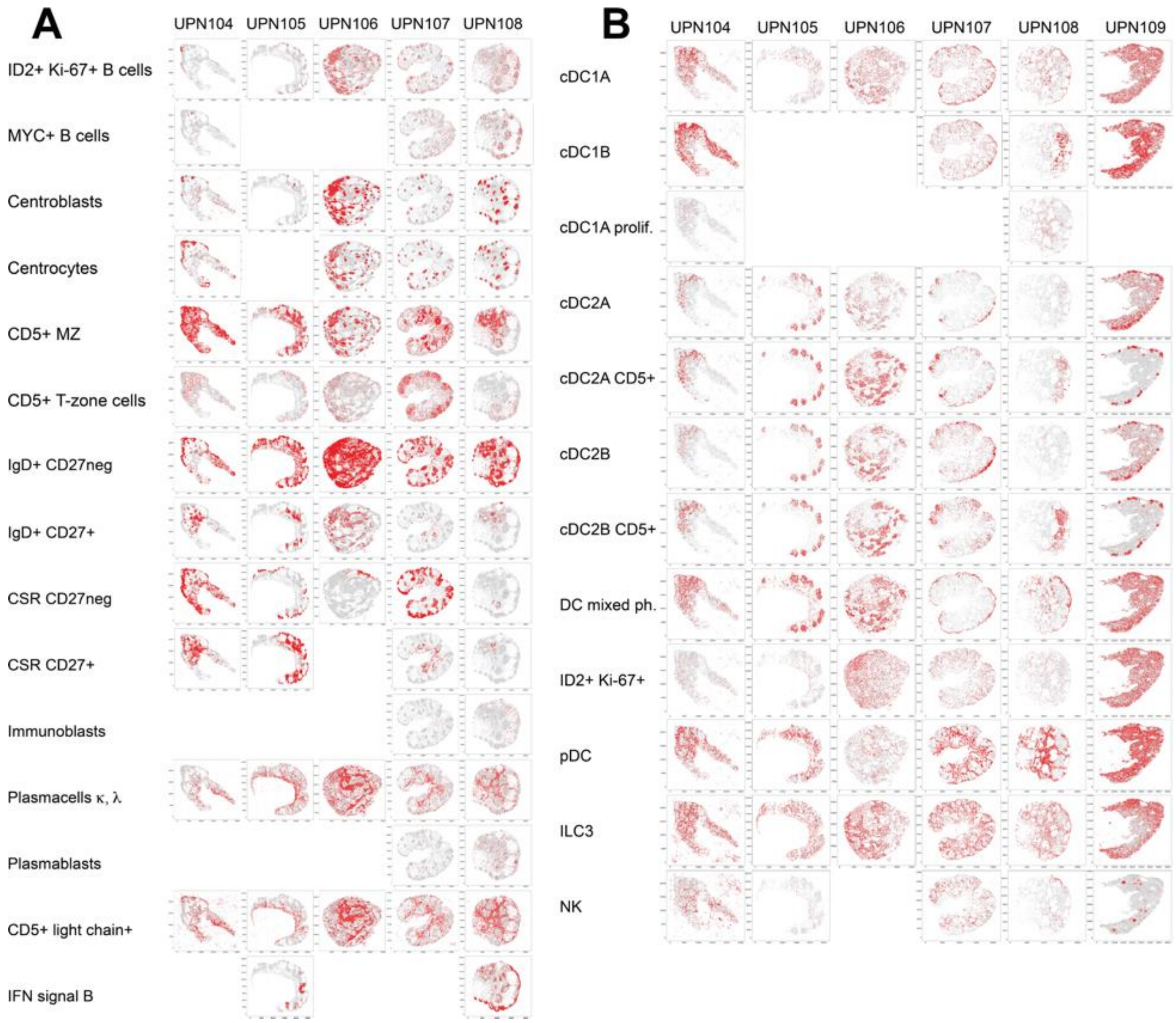

S4 Fig. (related to Fig 6 and 9). Spatial distribution of B-cell and dendritic cell subsets.
A: Spatial distribution of B-cell subsets on five whole LN. Each population, the sum of the individual clusters, is shown in red over a gray shadow representing all B-cells. Each dot is a magnified cell.
B: Spatial distribution of dendritic and non-myeloid innate immune cell subsets on six whole LN. Each population, the sum of the individual clusters, is shown in red over a gray shadow representing all dendritic cells. Each dot is a magnified cell.

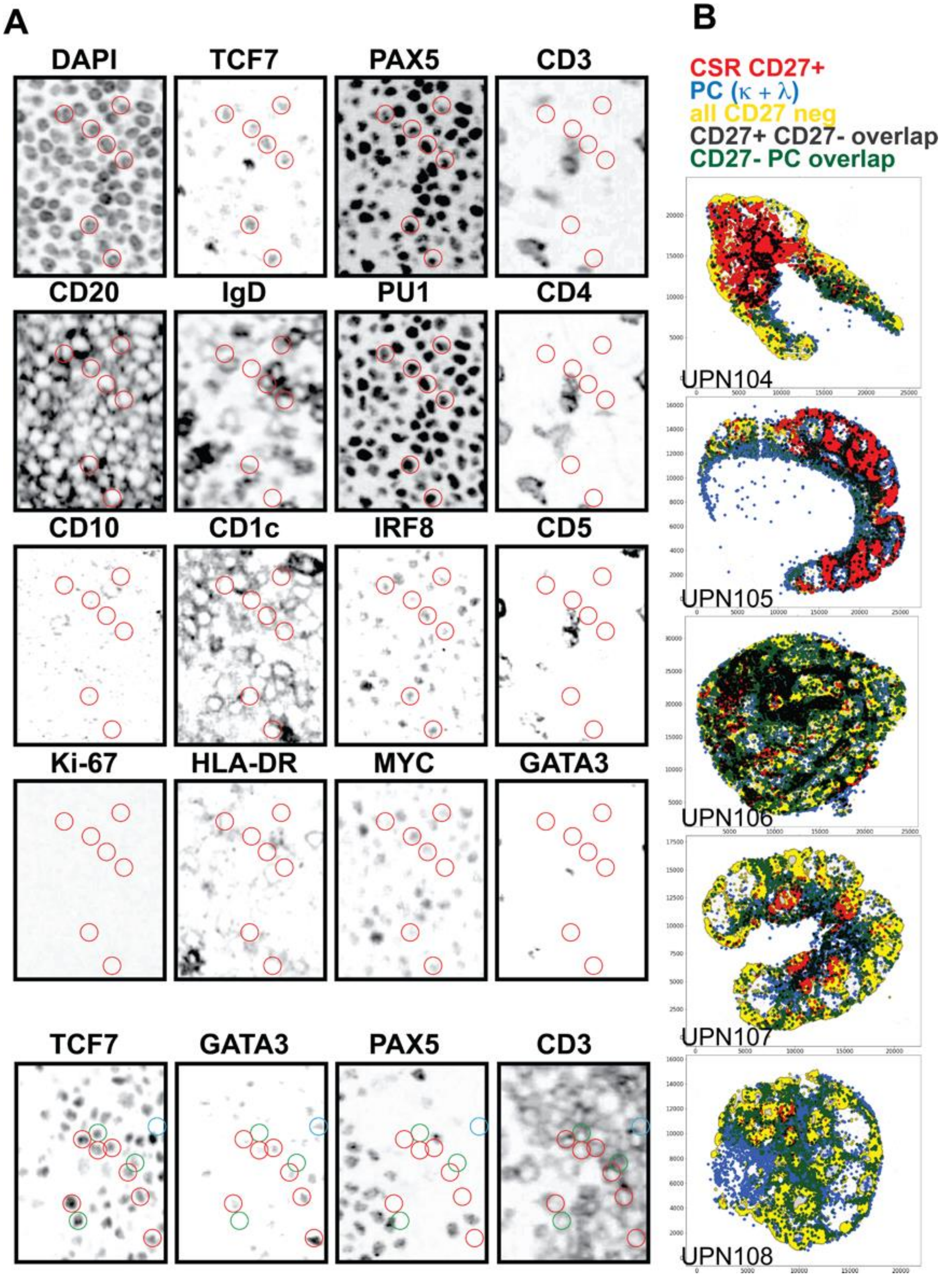

S5 Fig. (related to Fig 7). Multiplex phenotype and spatial allocation of CD5+ and memory B-cell subsets.

A: A: Multiplex phenotype of CD5+ MZ B cells coexpressing PAX5 and TCF7. The top 16 images show serial stainings for the same area from the marginal follicular border of LN UPN104, showing a coordinated TCF7+ immature transitional B cell phenotype, highlighted by

the circled cells. The bottom four serial images show a different area from the same LN close to a T-cell area, encompassing the border of a PAX5+ B cell follicle on the lower left and a T cell zone hosting numerous CD3+ T-cells coexpressing TCF7 and GATA3 (red circles), TCF7 but not GATA3 (green circles) and GATA3 but not TCF7 (blue circle). None of these T cells is PAX5+. The scale of each circle is 7 µm. The grayscale IF images have been inverted and adjusted for visibility.
B: Spatial allocation of CSR memory (CD27+), plasma cells (k + l) and all CD27neg B-cell subsets. CSR memory (CD27+; red), plasma cells (k + l; blue) and all CD27neg B-cell subsets (yellow) are allocated in the LN space. Single cells from each cell type represented as enlarged dots have been coalesced, the outline border darkened and superimposed in Adobe Photoshop. CSR memory B-cells and plasmacells show the least superimposition (dark violet), these latter showing greater overlap with naive B-cells (green) at the mantle zone border. Note that all CD27+ B-cell subsets do not have neighborhood relationships with plasma cells at the single cell level.

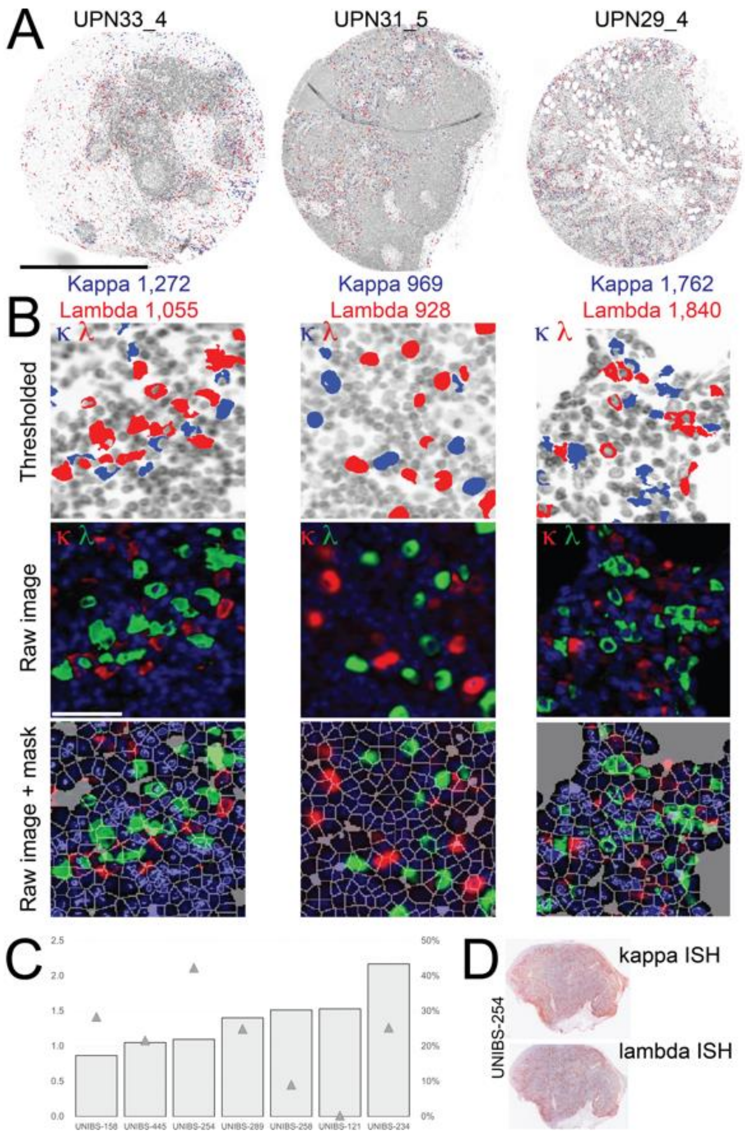

S6 Fig. (related to Fig 8). Validation of plasma cell identification by manual thresholding and comparison with ISH for light chains.

A: Three TMA cores are shown as inverted IF image of nuclei (grey, DAPI), kappa light chain+ plasma cells (blue), lambda+ PC (red). The number of PC in each core is shown color-coded at the bottom of each core. Scale bar: 1mm.

B: A representative field from A is enlarged for each TMA core. Top row: intensity thresholded kappa (blue) or lambda (red) PC are superimposed onto the DAPI inverted image (grey). Middle row: raw IF images of the same field is shown as a RGB composite with nuclear DAPI (blue), kappa (red) and lambda (green) PC. The PC count with CyBorgh for UPN33_4 is 1473 (kappa) and 1268 (lambda), with CellPose 1362 (kappa) and 862 (lambda). PC count (CyBorgh) for UPN31_5 are 1048 (kappa) and 1541 (lambda). For UPN29_4 are 934 (kappa) and 2779 (lambda). Bottom row: a segmentation mask (CyBorgh) is superimposed to the middle row IF composite.

C: The double-scale graph shows the kappa/lambda ratio for ISH for light chain RNA (grey bars, scale on the left). The triangles show the percentage of ISH-positive signals over the whole LN area (scale on the right).
D: low-power images of the ISH for light chain RNA on serial LN sections.

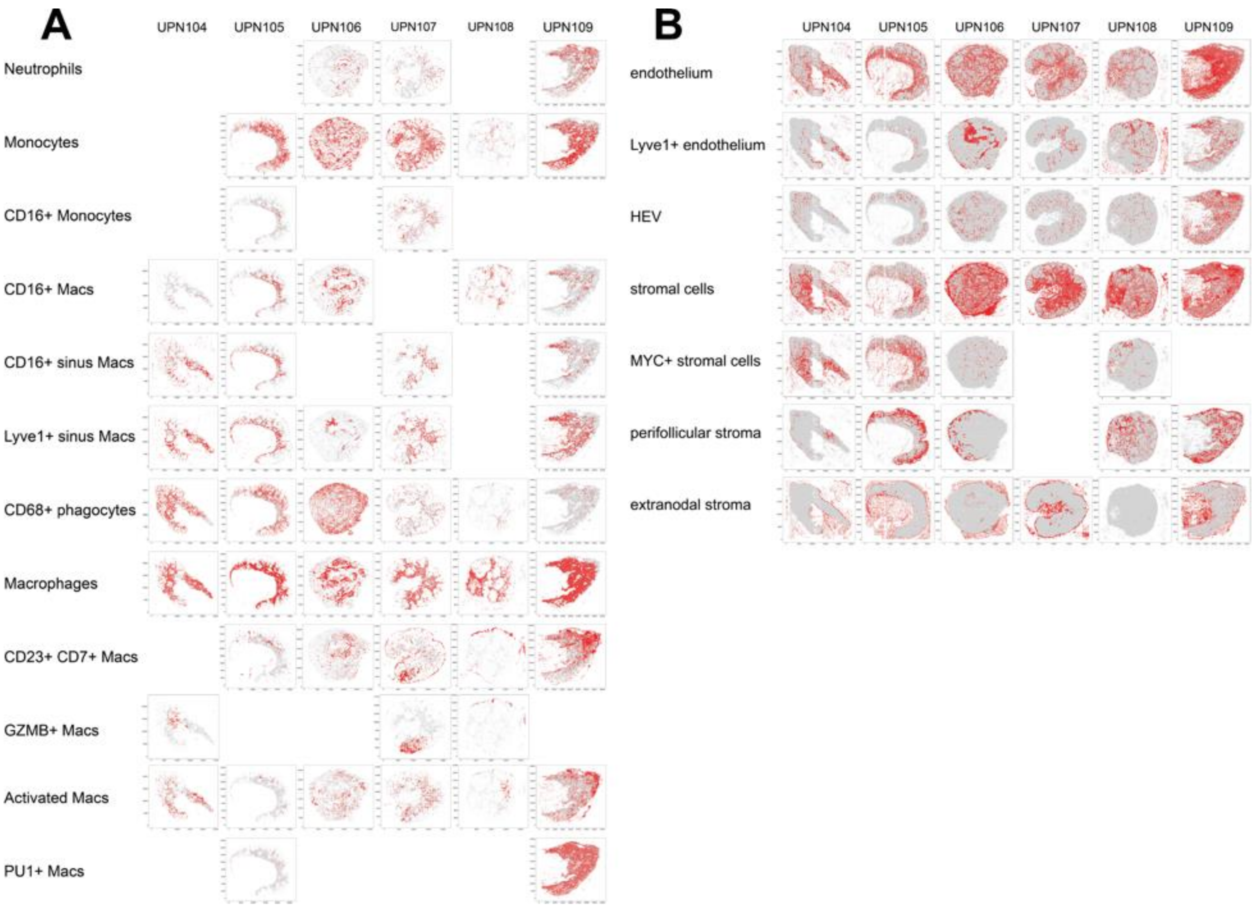

S7 Fig. (related to Fig 12 and to Fig 13). Spatial distribution of myeloid and stromal cell subsets.

A: Spatial distribution of myelomonocytic subsets on six whole LN. Each population, the sum of the individual clusters, is shown in red over a gray shadow representing all myelomonocytic cells. Each dot is a magnified cell

B: Spatial distribution of stromal cell subsets on six whole LN. Each population, the sum of the individual clusters, is shown in red over a gray shadow representing all stromal cells. Each dot is a magnified cell.

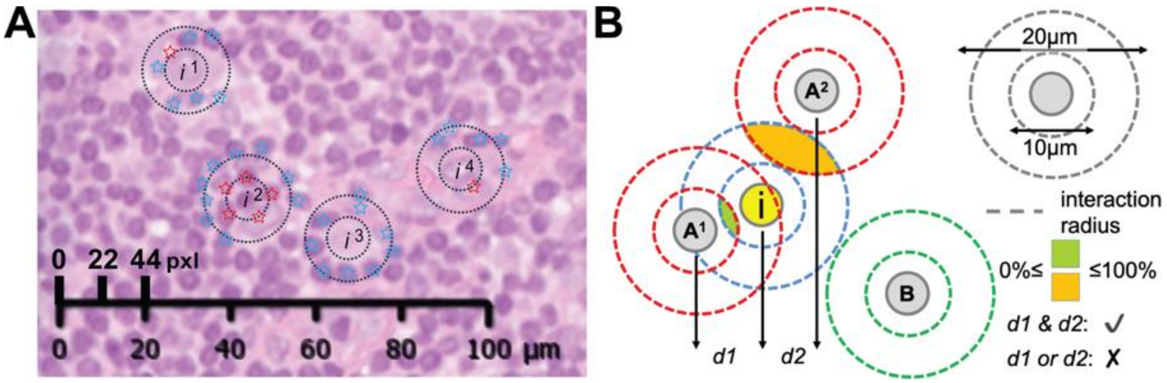

S8 Fig. (related to Fig 14). Global neighborhood relationships and spatial distribution overlap.

A: Neighborhood relationships at 22 and 44 pixels. A high magnification H&E image of a LN shows four index cells (in[superscript]) around whose center an inner 22 pixels circle and an outer 44 pixels circle are drawn. A red star marks neighboring nuclei encroached by the 22 pixel circle, a blue star the ones for the 44 pixel circle. Two macrophages (i1 and i3) contact one or no nuclei in the inner circle and 6 and 9 nuclei in the outer circle. A lymphocyte (i2) contacts five and nine nuclei in the inner and outer circle respectively. An endothelial cell (i4) contacts a single close neighboring nucleus and five nuclei in the outer circle. Scale: 0.45 µm per pixel.

B: Overlap scheme. By choosing an interacting area the overlap measure is the percentage of shared interacting areas among different cells. The overlap measure is bounded between 0% and 100%, and interactions were defined significantly only if 10 µm overlap (d1) AND 20 µm overlap (d2) resulted both significantly enhanced with respect to randomly placed cells (tested with bootstrap method). In this scheme, ***i*** is the index cell evaluated for overlap with two ***A*** cells belonging to a different cell type; those fulfill the statistical criteria for overlap, while the ***B*** cell does not.

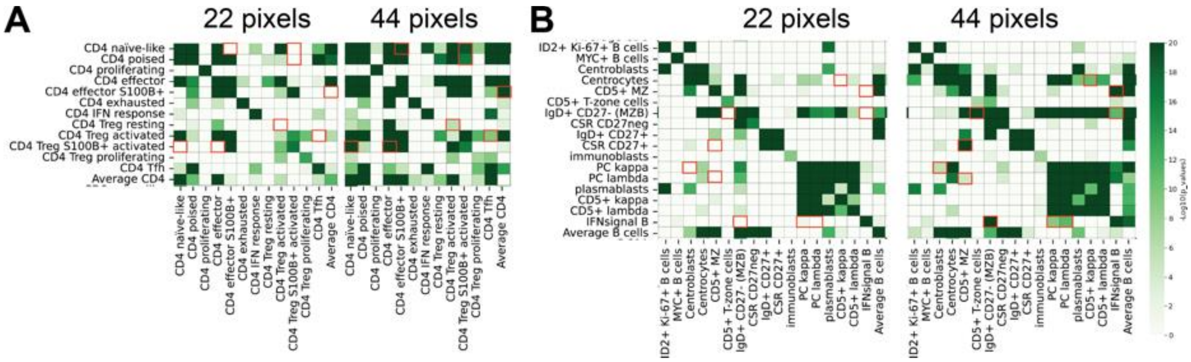

S9 Fig. (related to Fig 14). Examples of neighborhood relationships at 22 and 44 pixels for CD4 T-cells and B-cells.

Comparison of the significance of neighborhood relationships at 22 or 44 pixels for CD4+ T-cells (**A**) or B-cells (**B**). The figure contains all statistical significance values for each cell type (rows) versus the others (columns). Only the values equal or smaller than p 8.094544277157197e-06 (approx. 0.0000081) are shown. Color scale at the right. Statistically significant relationships at 22 pixels are in general captured and confirmed at 44 pixels as well, because of a larger capture area. However, significant relationships at 44 pixels only (red squares) can be observed and represent spurious results from passers-by cells. The neighborhood analysis for all cell types can be downloaded from UNIMIB Digital Commons repository Bicocca Open Archive Research Data (BOARD), DOI:10.17632/3ntbp3zdzh.2 1 pixel = 0.45 μm.

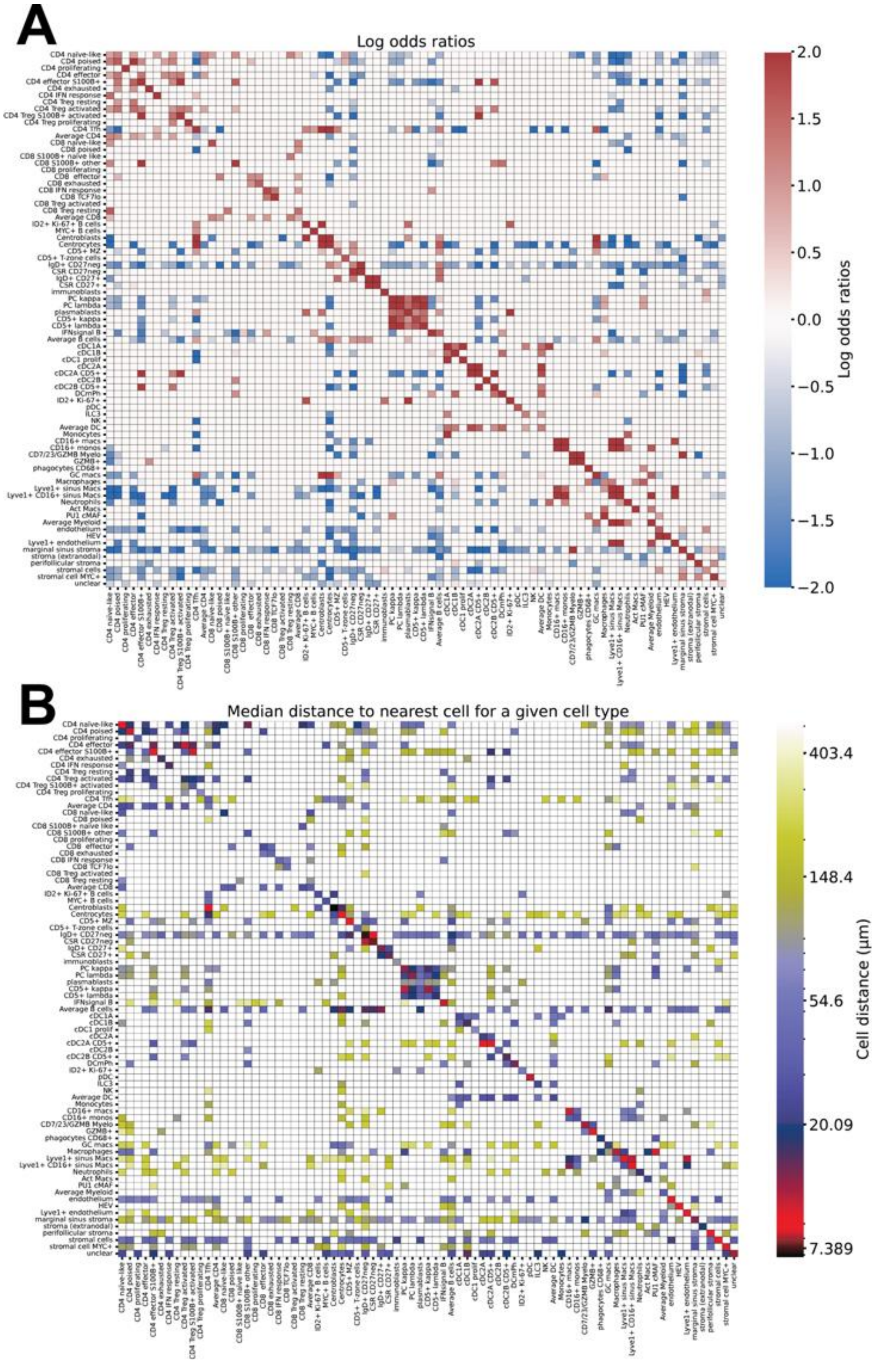

S10 Fig. (related to Fig 14). Global odds ratios and mean distance of nearest cells.
A: Logarithm of odds ratio obtained from Fisher exact test. Only the values equal or smaller than p 8.094544277157197e-06 (approx. 0.0000081) are shown, where in order to combine p values from different thresholds and different lymph nodes the maximum p value was taken to show only most robust results. Color scale at the right.

B: Median distance of each cell type (rows) to the nearest cell of a given type (columns).To aggregate the results from different threshold and different lymph nodes the median of medians was taken. Color scale at the right.

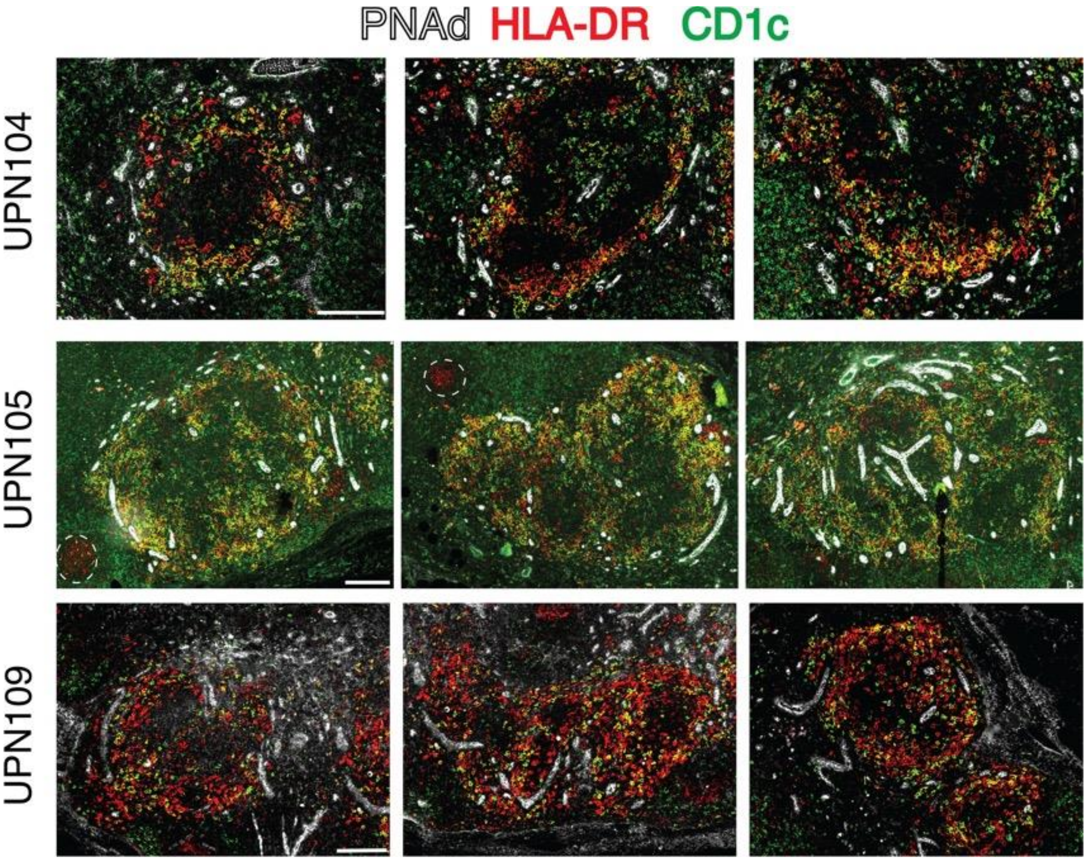

S11 Fig. (related to Fig 10). HEV and Fairy Circles spatial relationships.
Relationship between PNAd+ high endothelial venules (HEV) and Fairy Circles.
Three medium-power details from each of three lymph nodes are shown. The HEV are shown in white, the Fairy Circles are a commixtion of HLA-DR (red) and CD1c (green) positive cDC2 dendritic cells. Two small HLA-DR+ germinal centers are circled (dashed circle) in UPN105.
Space bar : 200 µm.

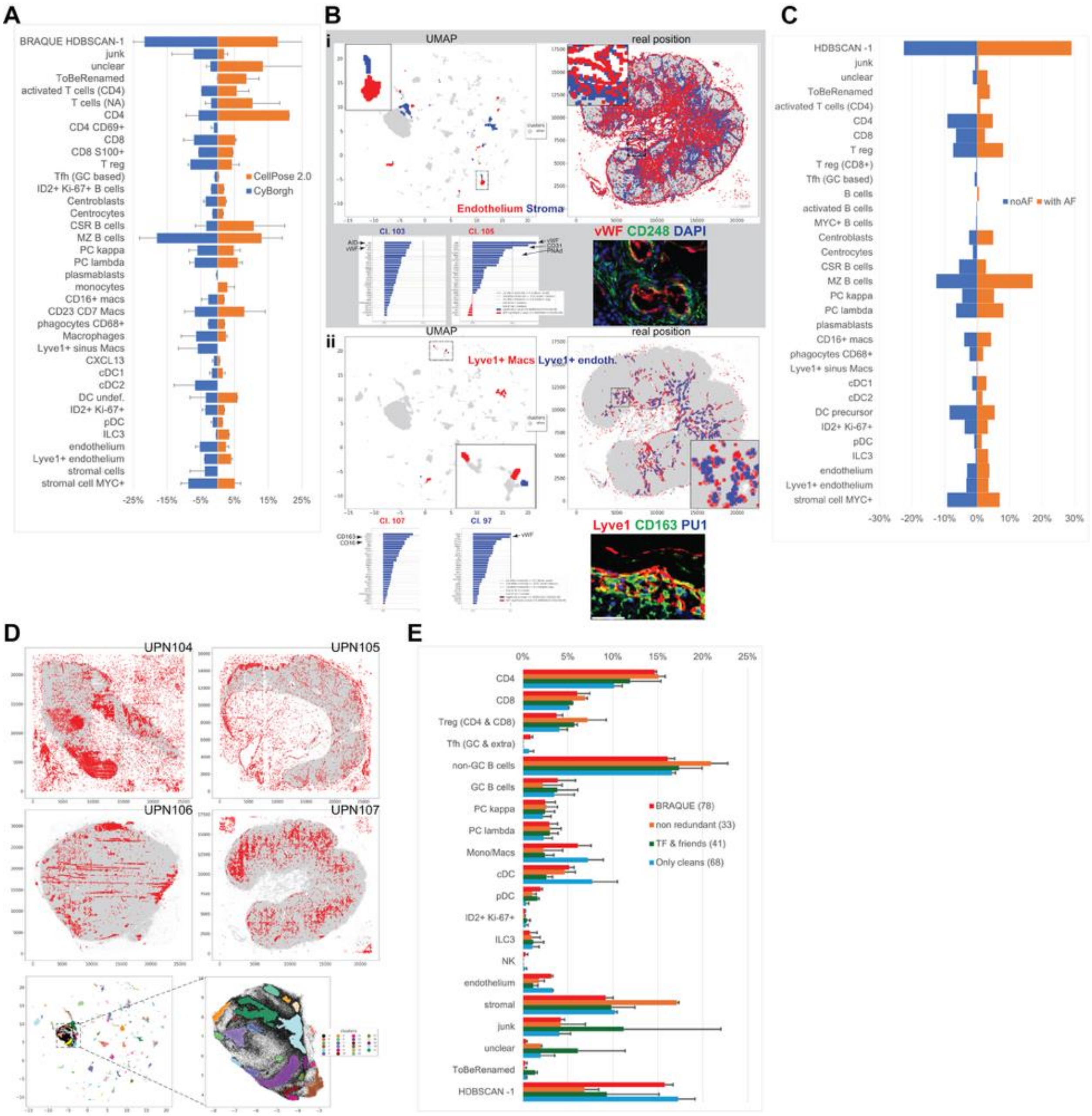

S12 Fig. (related to Fig 2). Validation of the BRAQUE pipeline analysis.

A: Comparison of image segmentation with Cellpose 2.0 and CyBorgh. Three TMA cores (UPN26, 32 and 33) were segmented using Cellpose 2.0 or CyBorgh (part of the BRAQUE pipeline). BRAQUE was run on the .csv data files and the clusters were classified. The mean cell type percentages ± SD are shown. For an explanation of the cell types, see S5 Table.

B: Spatial and phenotypic discrimination of juxtaposed cells by BRAQUE. Two examples of cells juxtaposed are shown both in the UMAP and in the real space for UPN107. In each panel the clusters of endothelium and stroma (i, gray background), Lyve1+ macrophages and Lyve1+ endothelium (ii, white background) are plotted in contrasting colors, respectively on the UMAP (left) and real space (right). Details are magnified as insets. The markers significance distribution of two representative clusters for each pair are shown below the UMAP maps.

Below each real space, a RGB composite of DAPI (blue) and color-coded representative markers for each cell type (red, green) are shown.
i: Endothelium (red) and stromal cells (blue) clusters are plotted in the UMAP space and on the real tissue space. The dashed squares are enlarged as inserts. The marker expression ranking of two adjacent clusters shown in the insert, clusters 103 (stromal) and cluster 105 (endothelial) is shown, with relevant markers highlighted. The triple IF RGB color image shows nuclear DAPI (blue), endothelial vWF (red) and fibroblasts CD248 (green). Note the very close relationship of CD248 fibroblasts with vWF+ endothelium. Scale bar: 500 μm.
ii: Lyve1+ Macrophages (red) and Lyve1+ endothelium (blue) clusters are plotted in the UMAP space and on the real tissue space. The dashed squares are enlarged as inserts. The marker expression ranking of two representative clusters, Lyve1+ Macrophages and Lyve1+ endothelium is shown, with relevant markers highlighted. The triple IF RGB color image shows nuclear macrophage PU1 (blue), endothelial Lyve1 (red) and macrophage CD163 (green). Note Lyve1+ and Lyve1- CD163+ macrophages displaying nuclear PU1. Scale bar: 100 μm
C: Comparison of cell classification by BRAQUE on AF-subtracted and raw images. 75 images from a TMA core (UPN32) were either autofluorescence-subtracted (noAF) or left untreated (with AF), then both were independently analyzed with BRAQUE and the clusters obtained were classified. The percentage for each cell type is reported.
D:Technical artifacts and excluded cells. Clusters classified as technical artifacts (“junk”) are plotted on real space. The scale represents pixels (0.45 μm/pixel). Bottom left: the color-coded clusters plotted on the UMAP space of UPN107. Black dots represent clusters/cells which are allocated in the HDBSCAN -1 cluster. The dotted area is magnified on the right.
E: Effect of the antibody panel composition on the detection of cell types by BRAQUE. Two LN (UPN107 and UPN108), stained with 78 markers and segmented, were analyzed with four panels: *i*) BRAQUE with the full panel (78 Abs), *ii*) non redundant, mutually exclusive markers (33 Abs), *iii*) a combination of anti-transcription factor antibodies and 14 key diagnostic markers (TF & friends; 41 Abs) and *iv*) antibodies selected for highest signal-to-noise ratio and cell type identification power (Only cleans; 68 Abs). The percentage of broad cell types in each panel is shown ±SD. Note the larger SD and an increase in junk and unclear clusters with the smaller panels. For details of the panel’s composition see S6 Table, S17 Table. S17 Table contains individual cases results with a detailed cell list.

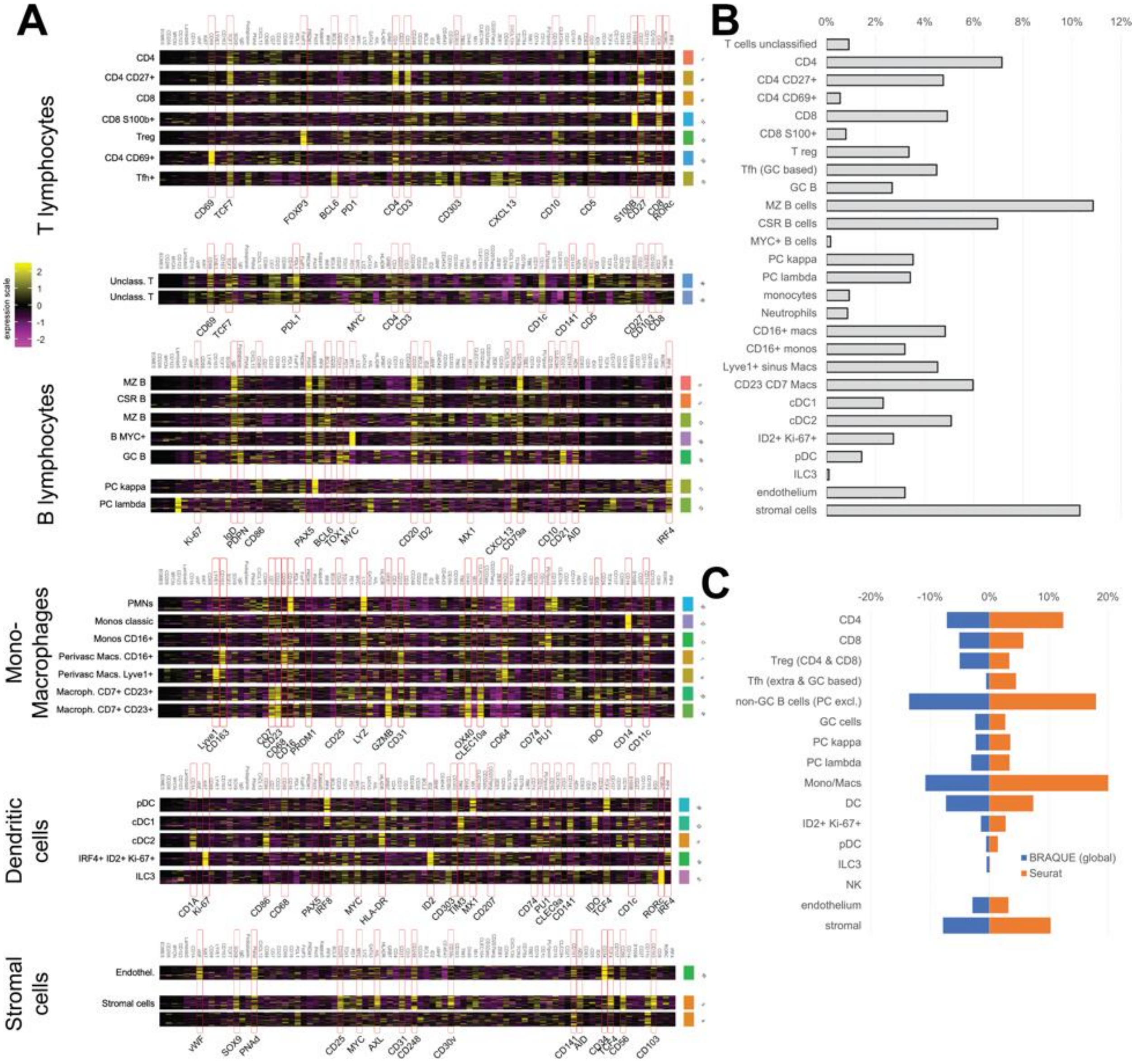

S13 Fig. (related to Fig 2). Cluster analysis (Seurat) of 37 normal lymph node Tissue Microarray cores.

A: 37 TMA cores from 18 subjects stained in multiplex are clustered with Seurat. Individual clusters are grouped by cell type classification. Relevant markers for each group are highlighted (red rectangle and marker name at the bottom). Cluster 45 to 51 have been removed because of insufficient cell numbers each. Unclassifiable clusters 36, 38, 42 and 43 have been removed.

B: Cell type composition of 37 TMA cores from 18 subjects are clustered with Seurat. Individual clusters are grouped by cell type classification and the percentage composition is plotted. Note that unclear clusters (199992 cells, 10.7% of the total) have been removed before calculating the percentage of each type. The cell type names are different from the analysis with BRAQUE and are assigned by manual scoring of the heatmaps.

C: Comparison of global BRAQUE analysis and Seurat. 37 TMA cores from 18 subjects stained in multiplex are clustered with Seurat and BRAQUE. The cell types from BRAQUE are grouped in broad cell types for the comparison.

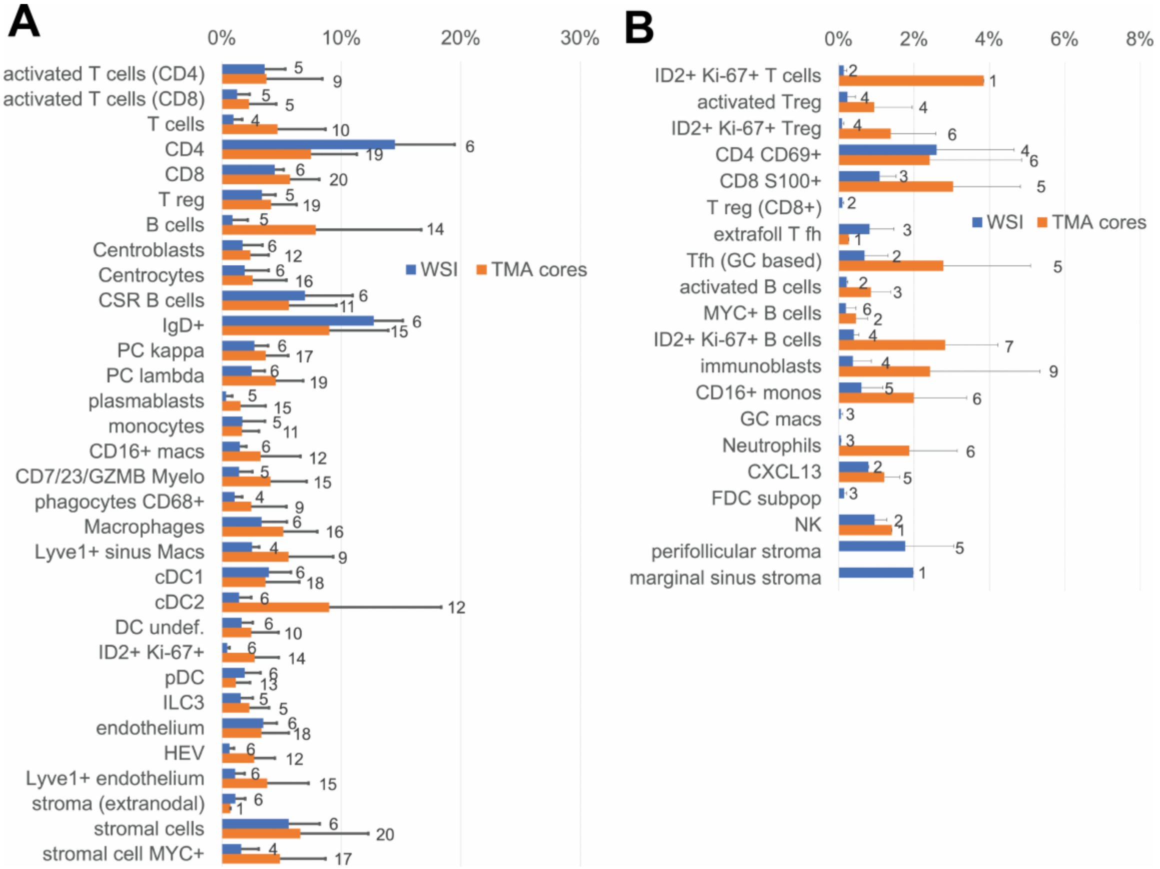

S14 Fig. (related to Fig 2). Comparison of cell type frequency in whole sections vs TMA cores.

Cell type frequency in whole sections and TMA cores identified in 27 LN samples (6 whole LN and 21 2mm TMA cores) after BRAQUE^{global} analysis on all cells in each sample, expressed as percentage ± SD. The cell types are separated into **A**: frequent cell types (i.e. ≥ 20 total clusters) and B: infrequent cell types (i.e. <20 total clusters). The numbers next to the bars are the number of informative cases for that cell type. Note frequency discrepancies, most notable in B, referable to the TMA area sampling selection. See also S3 Table.

SUPPLEMENTARY REFERENCES

1. Dall'Olio L, Bolognesi M, Borghesi S, Cattoretti G, Castellani G. BRAQUE: Bayesian Reduction for Amplified Quantization in UMAP Embedding. Entropy (Basel). 2023;25(2):354. Epub 20230214. doi: 10.3390/e25020354. PubMed PMID: 36832720; PubMed Central PMCID: PMCPMC9955093.
2. Bolognesi MM, Dall'Olio L, Maerten A, Borghesi S, Castellani G, Cattoretti G. Seeing or believing in hyperplexed spatial proteomics via antibodies: New and old biases for an image-based technology. Biol Imaging. 2024;4:e10. Epub 2024/10/23. doi: 10.1017/S2633903X24000138.
